# The effect of contextual factors on unintentional injury hospitalization: from the Korea National Hospital Discharge Survey

**DOI:** 10.1186/s12889-018-5249-4

**Published:** 2018-03-13

**Authors:** Hye Ah Lee, Hyejin Han, Seonhwa Lee, Bomi Park, Bo Hyun Park, Won Kyung Lee, Ju Ok Park, Sungok Hong, Young Taek Kim, Hyesook Park

**Affiliations:** 1 Clinical Trial Center, Mokdong Hospital, Ewha Womans University, Seoul, Republic of Korea; 2 Department of Preventive Medicine, College of Medicine, Ewha Womans University, 1071, Anyangcheon-ro, Yangcheon-ku, Seoul, 158-710 Republic of Korea; 3 Department of Social and Preventive Medicine, Inha University School of Medicine, Incheon, Republic of Korea; 4 Department of Emergency Medicine, Hallym University College of Medicine and Dongtan Sacred Heart Hospital, Hwaseong, Republic of Korea; 5 Division of Chronic Disease Control, Korea Centers for Disease Control and Prevention, Cheongju-si, Republic of Korea

**Keywords:** Contextual effect, Hospitalization, Multilevel analysis, Unintentional injury

## Abstract

**Background:**

It has been suggested that health risks are affected by geographical area, but there are few studies on contextual effects using multilevel analysis, especially regarding unintentional injury. This study investigated trends in unintentional injury hospitalization rates over the past decade in Korea, and also examined community-level risk factors while controlling for individual-level factors.

**Methods:**

Using data from the 2004 to 2013 Korea National Hospital Discharge Survey (KNHDS), trends in age-adjusted injury hospitalization rate were conducted using the Joinpoint Regression Program. Based on the 2013 KNHDS, we collected community-level factors by linking various data sources and selected dominant factors related to injury hospitalization through a stepwise method. Multilevel analysis was performed to assess the community-level factors while controlling for individual-level factors.

**Results:**

In 2004, the age-adjusted unintentional injury hospitalization rate was 1570.1 per 100,000 population and increased to 1887.1 per 100,000 population in 2013. The average annual percent change in rate of hospitalizations due to unintentional injury was 2.31% (95% confidence interval: 1.8–2.9). It was somewhat higher for females than for males (3.25% vs. 1.64%, respectively). Both community- and individual-level factors were found to significantly influence unintentional injury hospitalization risk. As community-level risk factors, finance utilization capacity of the local government and neighborhood socioeconomic status, were independently associated with unintentional injury hospitalization after controlling for individual-level factors, and accounted for 19.9% of community-level variation in unintentional injury hospitalization.

**Conclusion:**

Regional differences must be considered when creating policies and interventions. Further studies are required to evaluate specific factors related to injury mechanism.

## Background

Injuries result from traffic accidents, falls, poisonings, etc. and collectively result in the deaths of more than five million people worldwide each year [1]. Furthermore, injury is one of the leading causes of hospitalization and may also lead to disability, which can result in lower quality of life [2] and high medical costs. Consequently, injury is a major public health issue.

Although in Korea the mortality rate due to road traffic accidents has decreased significantly from 17.1 per 100,000 in 2004 to 11.9 per 100,000 population in 2013 [3], Korea ranks second after Austria in the number of hospitalizations due to injuries, poisoning, and external causes among Organization for Economic Co-operation and Development (OECD) countries (2755.8 and 3006.8 per 100,000 population in Korea and Austria, respectively, in 2013) [4].

To establish an effective intervention strategy, it is important to understand the scale of injury risk and identify the risk factors for injury. Accordingly, a systematic data collection system is required. Since 2004, the Korea Centers for Disease Control and Prevention (KCDC) has included In-Depth Injury Surveillance as a part of the Korea National Hospital Discharge Survey (KNHDS) to collect data from hospital-based injury surveillance systems [5].

The risk of unintentional injury is influenced by the environment in which an individual lives as well as by the individual’s characteristics (e.g. sex, age, etc.) and behaviors [6–8]. For these reasons, the Haddon matrix distinguishes environmental risk factors for injury [9]. In general, the impact of the individual-level factors means compositional effect, and the impact of the environment surrounding an individual means contextual effect. This is not limited to the physical environment, because people in the same geographic area also share a cultural, social, and economic environment. Consequently, health risks vary from area to area [10]. Thus, to better understand the influence of context on health outcomes, both community- and individual-level factors must be considered simultaneously. Other studies have reported the effects of community-level characteristics on individual health, but there is little evidence specifically regarding the effects on injury. There have been some studies that focused on fatal injuries [6, 7] or childhood injuries [6, 8, 11], or that did not consider various community-level factors.

This study focused on two major research questions. First, to understand the level of risk of unintentional injury, we investigated trends in unintentional injury hospitalization rates during the period from 2004 to 2013. We also assessed community-level risk factors for unintentional injury hospitalization after controlling for individual-level factors.

## Methods

### Data source and study subjects

The KNHDS has been conducted annually since 2004. The survey sampling was conducted based on clusters of hospitals stratified by geographic location and number of beds. The KNHDS data consisted of about 9% of discharge patients who were randomly sampled from among 170 sample hospitals with more than 100 beds (No. of participants = 214,569 for 2013 KNHDS) [12]. This survey included data from hospital admissions due to illnesses as well as those due to injuries. Injuries accounted for 17.5% (weighted percent, with considering sampling method) of hospital admissions in the 2013 KNHDS and 87.2% were unintentional injury. Data collected included each patient’s age, sex, residence zip code, type of insurance, and diagnostic code(s) of medical records (based on International Classification of Diseases 10th Revision). Additional data on injury intentionality and injury mechanisms recorded by doctors were collected. The injury mechanisms was defined as follows; transportation injury (ICD-10 V01-V99), falling, stumbling, jumping, pushed, etc. (W00-W19), contact with blunt force (W20-W24, W27-W31, W35-W40, W45, W49-W52, W54-W64), piercing, penetrating force (W25-W27, W29, W45-W49, W53, W54-W64), shot by firearm (W32-W34), thermal mechanism (X00-X19, X32), suffocation (W75-W84), drowning (W65-W74), exposure to chemical or other substance (X20-X29, X40-X49), other specified mechanism of injury (W35–44, W46, W49, W85-W99, X30-X39, X50-X58), unspecified mechanism of injury (X59) [12]. This study used data from the 2004 to 2013 KNHDS. Community level data was limited due to administrative district changes, so the 2013 KNHDS data were used to assess potential risk factors. In South Korea, the administrative divisions for lower-level local governments are Si/Gun/Gu. Thus, the community-level was classified as Si/Gun/Gu based on the residence zip codes of the patients. The study protocol was approved by the Institutional Review Board of Ewha Womans University Hospital.

### Data for individual-level factors

Age, sex, and type of insurance were collected as part of the KNHDS. Type of insurance was classified using data for payment of medical costs (national health insurance, medical aid, and vehicle insurance & others).

### Data for community-level factors

To find potential community-level risk factors, we used data from national institutions and surveys, which collected community-level data. Ultimately, 18 variables were considered in this study.

The data for financial independence ratio (FIR), proportion of welfare budget (social security) in general account, percentage of population which is elderly, number of fires per 10,000 residents, number of residents per 119 safety center, number of traffic accidents per 1000 cars, and the numbers of hospitals, nurses, and doctors were collected from the Korea National Statistical Office. Data for the numbers of hospitals, nurses, and doctors was only available for 2011, but was used regardless. The FIR is an index of the finance utilization capacity of a local government with independent discretionary power. Percentage of population that is elderly refers to the percentage of the population over 65 years of age.

The data for traffic safety index at the community level was obtained from the traffic accident analysis system [13]. The traffic safety index is an indicator for comparing and evaluating the level of traffic safety by quantifying the level of traffic safety in the community based on traffic accident statistics. Since 2005, it has been reported by the Korea Road Traffic Authority. The traffic safety index is expressed in percentiles, and a high value indicates a high level of safety in the community.

The Community Health Survey (CHS) provided other community-level indicators, including percentage of unemployed persons, percentage of persons who experienced depression, percentage of persons with perceived stress, percentage of seat belt use during driving, percentage of moderate physical activity, percentage of high-risk drinking, percentage of people with education less than a high school diploma, and percentage of National Basic Livelihood Security System (NBLSS) recipients. Percentage of moderate physical activity was defined as the percentage of people who have practiced intense physical activity for more than 20 min per day on more than 3 days per week or those who have practiced moderate physical activity for more than 30 min per day on more than 5 days per week during the last week. Percentage of high-risk drinking was defined as the percentage of people who drank in the last year who responded that they drank excessively (≥ 7 glasses for males and ≥5 glasses for females) more than twice a week. The CHS provides community health-related data to establish and evaluate community health plans. The detailed information about the CHS has already been published [14].

### Statistical analysis

In summary statistics, we presented age-adjusted unintentional injury hospitalization rates. They were estimated from a standard population based on data from the 2010 Census from the Korean National Statistical Office. The Joinpoint Regression Program was used to assess the trend of hospitalization rates due to unintentional injury. It can be freely downloaded from the National Cancer Institute homepage [15]. This program specifically tests for changes in trends. It also produces a graph for each joinpoint that exhibits any apparent change in trend. The change in hospitalization rate from 2004 to 2013 was calculated as: [(age-adjusted unintentional injury hospitalization rate in 2013 − age-adjusted unintentional injury hospitalization rate in 2004) / age-adjusted unintentional injury hospitalization rate in 2004] × 100%.

Community-level variables were summarized as median with interquartile range. The associations between age-adjusted unintentional injury hospitalization rate and community-level risk factors were assessed using the Spearman correlation.

To avoid multicollinearity and select the most influential community-level variables, a stepwise regression analysis was performed using the variables with significance of *p* <  0.1 in the correlation analysis. Then, multilevel analysis was performed to assess the effects of community-level variables while controlling for individual-level variables. In the multilevel analysis, unintentional injury hospitalization was considered the outcome variable. The control group was applied as a hospitalized person without any injury diagnosis. Individual-level variables were defined as level 1 and community-level variables were defined as level 2. First, we assessed a null model to determine the model’s validity for exploring the effects of contextual factors on hospitalization due to unintentional injury. Sequentially, level 1 factors, level 2 factors, and level 1 and level 2 factors were assessed. Sub-analyses using the same multilevel model, stratifying by sex and age groups, were also conducted. All analyses were conducted using SAS (ver. 9.4; SAS Institute, Cary, NC, USA) and Joinpoint Regression Program (Version 4.5.0.1—June 2017; Statistical Methodology and Applications Branch, Surveillance Research Program, National Cancer Institute, USA). A *p* value of < 0.05 was considered statistically significant using the two-tailed test.

## Results

**Fig. 1:**
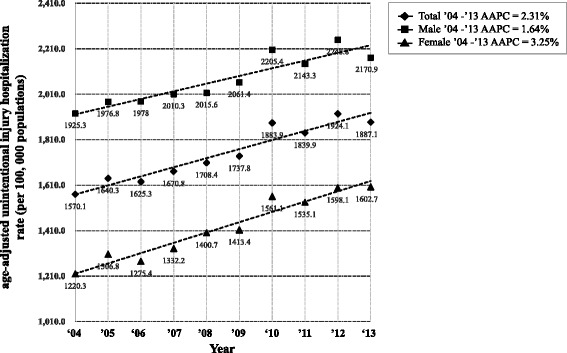
Average annual change (%) of age-adjusted unintentional injury hospitalization rate by sex. AAPC, average annual percent change. All AAPC results were significantly different from zero at alpha = 0.05. Standard population based on the 2010 Census data from the Korean National Statistical Office

In 2013, the age-adjusted unintentional injury hospitalization rate was 1887.1 per 100,000 population (male = 2170.9, female = 1602.7 per 100,000 population). The unintentional injury hospitalization rate has gradually increased since the survey began. The average annual percent change (AAPC) of the unintentional injury hospitalization rate was 2.31% (95% confidence interval [CI]: 1.8–2.9). The overall hospitalization rate was higher for males than for females, but the AAPC was higher for females (3.25%, 95% CI: 2.6–3.9) than for males (1.64%, 95% CI: 1.1–2.2). The difference was statistically significant (*p* <  0.01) (Fig. 1).

**Fig. 2:**
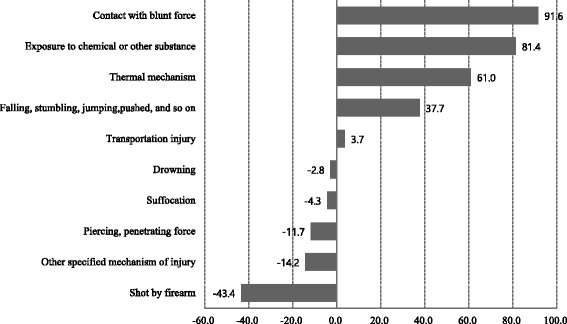
Change in hospitalization rate according to injury mechanism from 2004 to 2013. Unit:%. The change in age-adjusted hospitalization rate from 2004 to 2013 is calculated as follows: [(age-adjusted unintentional injury hospitalization rate in 2013 − age-adjusted unintentional injury hospitalization rate in 2004)/age-adjusted unintentional injury hospitalization rate in 2004] × 100%. Standard population based on the 2010 Census data from the Korean National Statistical Office

Traffic-related injuries were most common in 2004, but in 2013, falling injuries were with the most common due to a 37.7% increase. From 2004 to 2013, age-adjusted unintentional injury hospitalization rates due to falling among females increased 58.3%, from 463.5 to 733.9 per 100,000 population. Hospitalization rates due to contact with blunt force were the third most frequent and showed the greatest increase from 2004 to 2013. Exposure to chemical or other substances showed the second-greatest increase in hospitalization rate over the study period (Table 1, Fig. 2).

**Table 1:** Age-adjusted unintentional injury hospitalization rate by injury mechanism and surveillance year (per 100,000 population)

Injury mechanism	Total		Male		Female	
	2004	2013	2004	2013	2004	2013
Transportation injury	686.6	712.4	829.9	845.7	548.4	577.9
Falling, stumbling, jumping, pushed, etc.	518.1	713.4	570.9	693.7	463.5	733.9
Contact with blunt force	102.5	196.5	167.2	303.8	38.8	88.4
Piercing, penetrating force	74.3	65.6	114.0	94.2	34.9	36.7
Shot by firearm	0.4	0.2	0.8	0.5	0.0	0.0
Thermal mechanism	34.1	54.8	37.3	56.9	30.8	52.8
Suffocation	1.1	1.1	1.4	1.3	0.9	0.9
Drowning	1.1	1.1	1.5	1.5	0.7	0.6
Exposure to chemical or other substance	12.3	22.4	12.0	26.0	12.5	18.7
Other specified mechanism of injury	127.5	109.4	177.1	132.0	78.4	86.7
Unspecified mechanism of injury	32.1	70.8	39.2	85.3	25.0	56.4

Standard population based on the 2010 Census data from the Korean National Statistical Office

Table 2 shows the correlation coefficient between age-adjusted unintentional injury hospitalization rate and community-level variables along with a descriptive summary of community-level variables. The distribution of community-level variables varied across districts. Percentage of people with education less than a high school diploma, number of fires per 10,000 residents, and number of residents per 119 safety centers were more highly correlated with unintentional injury rates than other variables. Community-level financial ability and medical resources such as the number of hospitals, nurses, and doctors were negatively correlated with the age-adjusted unintentional injury hospitalization rate. In addition, the age-adjusted unintentional injury hospitalization rate was inversely correlated with the percentage of seat belt use during driving and the community-level traffic safety index. However, the number of traffic accidents per thousand cars was not correlated with unintentional injury hospitalization rate.

**Table 2:** Correlation between community-level variables and age-adjusted unintentional injury hospitalization rate in 2013

Community-level variables	N	Median	25th percentile	75th percentile	Spearman correlation (*ρ*)	*p* value
Financial independence ratio (%)	249	64.6	58.8	69.0	−0.11	0.07
Proportion of welfare budget (social security) in general account	249	27.1	16.8	39.8	− 0.19	< 0.01
Percentage of elderly population	249	13.9	10.1	22.6	0.19	< 0.01
Percentage of people with education less than a high school diploma	245	25.0	21.2	30.7	0.25	< 0.0001
Percentage receiving national basic livelihood security system	245	2.5	1.8	3.4	0.14	0.02
Number of hospitals in 2011	248	90.0	30.5	238.5	−0.22	< 0.01
Number of nurses in 2011	249	325.0	95.0	1119.0	−0.15	0.02
Number of doctors in 2011	249	217.0	66.0	632.0	−0.19	< 0.01
Number of fires per 10,000 residents	249	9.5	6.0	15.8	0.25	< 0.0001
Number of residents per 119 safety center	246	45,169.5	25,168.8	75,750.1	−0.25	< 0.0001
Traffic safety index	247	75.8	70.0	79.1	−0.17	< 0.01
Percentage of high risk drinking	243	28.0	24.7	31.4	0.13	0.04
Percentage of moderate physical activity	245	21.8	18.4	27.5	0.20	< 0.01
Percentage of seat belt use during driving	245	77.0	68.2	84.4	−0.18	< 0.01
Number of traffic accidents per thousand cars	249	9.4	8.0	10.8	0.03	0.59
Percentage of stress perception	245	26.6	23.4	29.5	−0.15	0.02
Percentage of experience of depression	245	5.2	4.0	6.7	−0.15	0.02
Percentage of unemployed persons	245	29.9	24.8	32.1	−0.23	< 0.001

Table 3 presents the results from the multilevel models of the estimated effects of individual- and community-level variables on the risk of unintentional injury hospitalization. Variation across districts was significant (Null model), and individual-level variables accounted for 19.2% of the community-level variation in unintentional injury hospitalization. When controlling for individual-level variables, community-level variables explained 19.9% of the community-level variation in unintentional injury hospitalization. Based on the individual-level model, the risk of unintentional injury hospitalization was 1.3 times (95% CI: 1.29–1.37) higher for males than for females. For each 5 years of increasing age, the risk of unintentional injury hospitalization increased by 3% (95% CI: 1.03–1.03). The effects of community-level variables remained constant in the multilevel model. The higher a district’s FIR, the lower the risk of unintentional injury hospitalization. Similarly, as the percentage of people with education less than a high school diploma increased, the risk of unintentional injury hospitalization also tended to increase. A higher percentage of high risk drinking in the community also tended to increase the risk of unintentional injury hospitalization, but it was not statistically significant.

**Table 3:** Multilevel analysis results for risk of unintentional injury hospitalization

	Null	Individual-level	Community-level	Multi-level
Fixed effect intercept (*β*)	−2.06 (*p* < 0.0001)	0.504 (*p* < 0.0001)	−2.214 (*p* < 0.0001)	0.195 (*p* = 0.31)
Male (ref. female)		**1.33** **(1.29, 1.37)**		**1.34** **(1.30, 1.38)**
Age (per 5 years)		**1.03** **(1.03, 1.03)**		**1.03** **(1.02, 1.03)**
Type of insurance (ref. national health insurance)				
Medical aid		**0.93** **(0.87, 0.99)**		**0.93** **(0.87, 0.99)**
Vehicle insurance & others		**28.71** **(27.44, 30.04)**		**28.17** **(26.89, 29.50)**
Financial independence ratio			**0.994** **(0.989, 0.998)**	**0.995** **(0.991, 0.999)**
Percentage of people with education less than a high school diploma			**1.010** **(1.001, 1.018)**	**1.009** **(1.001, 1.017)**
Percentage of moderate physical activity			**1.010** **(1.003, 1.018)**	**1.012** **(1.006, 1.018)**
Percentage of high risk drinking			1.002 (0.992, 1.012)	1.003 (0.995, 1.012)
Random effect (variance)				
Community	0.146 (*p* < 0.0001)	0.118 (*p* < 0.0001)	0.124 (*p* < 0.0001)	0.095 (*p* < 0.0001)

Bold results indicate *p* < 0.05

When stratified by sex and age group, the statistical significance of community-level effects were weakened and some showed borderline significance, but there was no difference in direction and size. However, the individual-level effects changed more substantially (Table 4).

**Table 4:** Sub-analysis by sex and age groups in the multilevel models

	Sex		Age groups		
	Male	Female	≤14 years	15–64 years	≥65 years
Fixed effect intercept (*β*)	0.802 (*p* < 0.0001)	−0.250 (*p* = 0.27)	−2.335 (*p* < 0.0001)	0.638 (*p* < 0.01)	−1.319 (*p* < 0.0001)
Individual- level					
Male (ref. female)	–	–	**1.545** **(1.389, 1.718)**	**2.099** **(2.017, 2.183)**	**0.551** **(0.519, 0.586)**
Age (per 5 years)	**0.988** **(0.98, 0.992)**	**1.094** **(1.088, 1.100)**	**2.583** **(2.438, 2.736)**	**0.944** **(0.938, 0.951)**	**1.157** **(1.130, 1.184)**
Type of insurance (ref. national health insurance)					
Medical aid	**0.864** **(0.787, 0.948)**	0.918 (0.838, 1.006)	0.918 (0.695, 1.213)	**0.800** **(0.730, 0.877)**	**0.883** **(0.798, 0.975)**
Vehicle insurance & others	**25.43** **(23.94, 27.01)**	**37.05** **(34.40, 39.91)**	**15.44** **(12.74, 18.71)**	**25.51** **(24.16, 26.93)**	**29.08** **(25.76, 32.82)**
Community-level					
Financial independence ratio	**0.996** **(0.992, 0.999)**	**0.995** **(0.990, 0.999)**	0.998 (0.992, 1.004)	0.996 ^a^ (0.991, 1.000)	0.996 ^a^ (0.991, 1.000)
Percentage of people with education less than a high school diploma	**1.009** **(1.001, 1.017)**	1.009^a^ **(**1.000**,** 1.019**)**	1.014 ^a^ **(**1.000**,** 1.029**)**	**1.013** **(1.004, 1.022)**	1.008 ^a^ **(**1.000**,** 1.017**)**
Percentage of moderate physical activity	**1.012** **(1.006, 1.019)**	**1.011** **(1.003, 1.019)**	1.002 (0.990, 1.014)	**1.014** **(1.006, 1.021)**	**1.012** **(1.005, 1.019)**
Percentage of high risk drinking	1.002 (0.993, 1.011)	1.005 (0.994, 1.016)	1.000 (0.985, 1.016)	1.004 (0.993, 1.014)	1.003 (0.994, 1.013)
Random effect (variance)					
Community	0.0831 (*p* < 0.0001)	0.114 (*p* < 0.0001)	0.095 (*p* < 0.0001)	0.112 (*p* < 0.0001)	0.0722 (*p* < 0.0001)

Bold results indicate *p* < 0.05

^a^*p* value was 0.5 ≤ *p* < 0.7

## Discussion

From 2004 to 2013, the hospitalization rate due to unintentional injury showed a linear trend, with an average annual increase of 2.31% (1570.1 per 100,000 population in 2004 and 1887.1 per 100,000 population in 2013, respectively). It was somewhat higher for females (3.25%) than for males (1.64%). Traffic accident-related; falling, stumbling, jumping, and being pushed; and contact with blunt force were in the top three types of injury in the survey years of 2004 and 2013. Of the community-level risk factors, FIR, percentage of low-level education status, and physical activity were independently associated with unintentional injury hospitalization, even when controlling for individual-level factors.

Although the injury mortality rate has decreased from 62.9 per 100,000 in 2004 to 61.3 per 100,000 in 2013 [3], hospitalization has increased. According to a study from the Korean Burden of Disease Study 2012, the proportion of the years lived with disability (YLD) to the disability-adjusted life year (DALYs) was higher than the years of life lost (YLL) (61.8% and 38.2%, respectively) [2]. In addition, in the Global Burden of Disease study 2013, Korea (3136 DALYs per 100,000, 95% uncertainty interval (UI): 2680–3558) had a higher burden of injury than Australia (1984 DALYs per 100,000, 95%UI: 1717–2307) and Japan (2527 DALYs per 100,000, 95%UI: 2185–2953) in the Asia-Pacific region [16]. Accordingly, national interest and policies are needed to reduce the overall injury risk. The comparatively high increasing trend of unintentional injury hospitalization rate in women is likely to continue, due to aging in accordance with increased life expectancy. Thus, strategies must be established to reduce the burden due to injuries corresponding to changes in population structure. In particular, it is necessary to focus on the relatively large increase in injury mechanism compared with the past, along with the absolute injury hospitalization scale.

Our results were in line with Pickett and Pearl’s conclusion [17], which reported that neighborhood socioeconomic effects are generally modest and smaller than the individual-level effects. Although the magnitude of the contextual effect on unintentional injury hospitalization is not large, it explained 19.9% of the community-level variation in unintentional injury hospitalization. One study by Lee et al. [7] indicated that individual-level variables explained 95.2% of community-level variation in mortality (aged 35 years and over) due to injury, while community-level variables contributed only 1.1%. This discrepancy might be induced by outcome definition or study method. Lee et al. also reported that the contribution of community-level variables varied according to the injury mechanism [7]. Studies investigating injury mechanism, considering specific neighborhood factors such as legislated school zones, are needed.

In this study, the risk of unintentional injury hospitalization increased by 1% as the percentage of people with low-education status in the community increased. Education reflects the knowledge-related assets of a person and is considered to be one of the factors in socioeconomic status [18]. In addition, education is one of the measures in the deprivation index, which reflects neighborhood social status [7, 19]. Although it did not focus on hospitalization, a prospective study showed that a high proportion of people with a low level of education in a particular neighborhood was associated with increased risk of death due to traffic accidents, homicide, and other external causes even after adjusting for individual factors. Low family incomes, high poverty, and high proportions of crowded housing in a neighborhood were also independently associated [19]. In addition, the health effects of the individual-level low education level were associated with mortality due to all injury [7] and traffic accidents, homicide, and other external causes [19].

A study conducted in New South Wales, Australia studied the relationship between child unintentional injury and relative socioeconomic disadvantage. Children who lived in more disadvantaged areas were more likely than children in the least disadvantaged areas to be hospitalized due to traffic accident-related injuries, fires and burns, and poisoning, but not due to fall-related injuries [20]. A Canadian report also indicated that the injury hospitalization rate tended to decrease as neighborhood income increased. This pattern was similar for all types of injury and for assault-related injuries [21], but it was not controlled for individual-level factors.

In this study, FIR also assessed the financial capacity of the community, which may be related to the installation of local safety facilities and implementation of health programs. Another Korean study showed that FIR negatively correlated with overall mortality for both sexes [22] and communities with smoke-free ordinances showed relatively higher FIR than did their counterparts [23]. However, one study reported a null association between FIR and fatality due to severe injury [24]. While investigating contextual effects, each study applied specific variables, so it is difficult to compare results. The positive association between hospitalization and moderate physical activity at the community-level seems to be the result of not only sports activities but also physical labor. Therefore, it seems necessary to consider the expansion of safety facilities in the playground and the workplace as a preventive strategy.

This study has some limitations. First, the control population was not healthy individuals but other hospital patients. Although hospital controls may share common risk factors, the community-level factors were independently associated with risk of unintentional injury hospitalization. The definition of a geographical area derived from a zip code may not be exactly the same as the location where the accident occurred, especially in the case of traffic accidents. Therefore, it will be necessary to consider the area in which the accidents occurred as opposed to the area of residence in future studies. We have not considered various individual-level variables due to the scope of data collection. In addition, there were limits to the data collected at the community level. These limitations are natural because the above data was not collected for research purposes. However, there is a need to improve the surveillance system for more specific data collection. Finally, confounding effects caused by unmeasured factors may remain.

Nevertheless, this study tried to identify relevant community-level factors by linking various data sources. Most of the data sources focused on socioeconomic factors. Our study considered community-level medical resources, financial capacity, and behavioral factors of residents as well as socioeconomic factors. In an ecological approach, the age-adjusted unintentional injury hospitalization rate was significantly correlated with the above factors. However, to understand the role of the contextual effect, both community- and individual-level factors must be considered together [10, 17]. By that standard, our results were produced by applying appropriate statistical methods. In addition, policy planning and preventive interventions are generally implemented on the basis of geographical area, taking into account community characteristics. Therefore, research on the determinants of contextual effects can provide useful information for policy making. Finally, we used a representative data source for hospitalization.

## Conclusions

In conclusion, this study assessed the trend of injury hospitalization rate over the past decade and found that it was steadily increasing. In addition, through multilevel analysis, our results showed that, along with regional differences in the risk of unintentional injury hospitalization, several community-level factors were dominant contextual effects. Therefore, there is a need for efforts to reduce regional health inequalities, with an understanding of the regional differences in injury risk. In particular, safety education opportunities should be provided to people with low socioeconomic levels. Although further studies are needed to consider specific factors (e.g. legislated school zones and participation rate in fire safety education, etc.) related to injury mechanism, this study is meaningful in that it assessed contextual factors for overall unintentional injury risk.

## Abbreviations

AAPC: Average annual percent change
CHS: Community Health Survey
CI: Confidence interval
DALYs: Disability-adjusted life year
FIR: Financial independence ratio
ICD-10: International classification of diseases 10th revision
KCDC: Korea Centers for Disease Control and Prevention
KNHDS: Korea National Hospital Discharge Survey
NBLSS: National Basic Livelihood Security System
OECD: Organization for Economic Co-operation and Development
YLD: Years lived with disability
YLL: Years of life lost
